# Combination of Trabectedin and Gemcitabine for Advanced Soft Tissue Sarcomas: Results of a Phase I Dose Escalating Trial of the German Interdisciplinary Sarcoma Group (GISG)

**DOI:** 10.3390/md13010379

**Published:** 2015-01-13

**Authors:** Bernd Kasper, Peter Reichardt, Daniel Pink, Michaela Sommer, Monika Mathew, Geraldine Rauch, Peter Hohenberger

**Affiliations:** 1 University of Heidelberg, Mannheim University Medical Center, Interdisciplinary Tumor Center Mannheim, Sarcoma Unit, Theodor-Kutzer-Ufer 1-3, D-68167 Mannheim, Germany; E-Mails: michaela.sommer@umm.de (M.S.); monika.mathew@umm.de (M.M.); peter.hohenberger@umm.de (P.H.); 2 HELIOS Klinikum Berlin-Buch, Sarcoma Center Berlin-Brandenburg, Schwanebecker Chaussee 50, D-13125 Berlin, Germany; E-Mail: peter.reichardt@helios-kliniken.de; 3 HELIOS Klinikum Bad Saarow, Pieskower Straße 33, D-15526 Bad Saarow, Germany; E-Mail: daniel.pink@helios-kliniken.de; 4 University of Heidelberg, Institute of Medical Biometry and Informatics, Im Neuenheimer Feld 305, D-69120 Heidelberg, Germany; E-Mail: rauch@imbi.uni-heidelberg.de

**Keywords:** gemcitabine, pazopanib, phase I, safety, soft tissue sarcoma, trabectedin

## Abstract

Background: Evaluation of the potential efficacy and safety of combination therapies for advanced soft tissue sarcomas (STS) has increased substantially after approval of trabectedin and pazopanib. Trabectedin’s introduction in Europe in 2007 depended mainly on its activity in so-called L-sarcomas (liposarcoma and leiomyosarcoma); combination of trabectedin with other chemotherapies used in STS seems of particular interest. Methods: We initiated within the German Interdisciplinary Sarcoma Group (GISG) a phase I dose escalating trial evaluating the combination of trabectedin and gemcitabine in patients with advanced and/or metastatic L-sarcomas (GISG-02; ClinicalTrials.gov NCT01426633). Patients were treated with increasing doses of trabectedin and gemcitabine. The primary endpoint was to determine the maximum tolerated dose. Results: Five patients were included in the study. Two patients were treated on dose level 1 comprising trabectedin 0.9 mg/m^2^ on day 1 and gemcitabine 700 mg/m^2^ on days 1 + 8, every 3 weeks. Due to dose-limiting toxicity (DLT) in both patients (elevated transaminases and thrombocytopenia), an additional three patients were treated on dose level −1 with trabectedin 0.7 mg/m^2^ plus gemcitabine 700 mg/m^2^. Of these three patients, two demonstrated another DLT; therefore, the trial was stopped and none of the dose levels could be recommended for phase II testing. Conclusion: The GISG-02 phase I study was stopped with the conclusion that the combination of gemcitabine and trabectedin is generally not recommended for the treatment of patients with advanced and/or metastatic leiomyosarcoma or liposarcoma. Also, this phase I study strongly supports the necessity for careful evaluation of combination therapies.

## 1. Introduction

Soft tissue sarcomas (STS) are a heterogeneous group of rare malignancies characterized by their mesenchymal origin and their poor prognosis. Around 50% of all patients diagnosed with STS will develop metastases at some point in the history of their disease. The most active drugs, doxorubicin and ifosfamide, achieve response rates around 15%–20% [1,2]. The limited efficacy of systemic treatment options results in a poor median overall survival for STS patients of about 12 months [3]. The introduction of new agents into the treatment armamentarium such as trabectedin [4] and pazopanib [5] may improve these results and suggests that longer survival could be achieved. With the aim to further improve patients’ survival, a combination of the new drugs with conventional chemotherapies used in STS has become a focus of clinical research.

The so called L-sarcomas represent about one fourth of all adult STS (15% liposarcomas and 11% leiomyosarcomas) [6]. Approval of trabectedin after treatment failure with anthracyclines and/or ifosfamide depended mainly on its activity in these L-sarcomas [7,8]. On the other hand, significant activity has been described for the use of gemcitabine and especially for the combination of gemcitabine and docetaxel, mainly in leiomyosarcomas [9]. The Sarcoma Alliance for Research through Collaboration (SARC) performed a randomized phase II study evaluating the efficacy of the combination of gemcitabine and docetaxel *versus* gemcitabine alone. This study favoured the combination with an increased response rate of 16% *versus* 9% for gemcitabine alone [10]. However, the combination of gemcitabine and docetaxel is associated with significant toxicity; pulmonary toxicity and refractory peripheral oedema are the most common severe adverse events. Thrombocytopenia and neutropenia are common, even with the use of growth factor support. Based on these data, the combination of trabectedin and gemcitabine became of interest, also for a possible later randomized comparison *versus* gemcitabine and docetaxel. Until now, safety and efficacy data using the combination of trabectedin and gemcitabine is scarce. One phase I trial evaluated weekly trabectedin and gemcitabine in patients with advanced solid tumors. The study demonstrated no pharmacokinetic interaction, and the recommended phase II dose for trabectedin was 0.4 mg/m^2^ and for gemcitabine 1000 mg/m^2^ for the weekly schedule. Seven out of 15 patients maintained stable disease after two cycles [11]. To formally evaluate the safety and efficacy of a possible combination treatment using trabectedin and gemcitabine, we initiated within the German Interdisciplinary Sarcoma Group (GISG) a phase I dose escalating trial in patients with advanced and/or metastatic L-sarcomas (GISG-02).

The aim of the present paper is to present the results of this trial which was stopped due to DLT as well as to discuss toxicities of combining new agents such as trabectedin and pazopanib with established chemotherapies for the treatment of advanced and metastatic STS.

## 2. Methods

### 2.1. Patient Population

Patients ≥18 years of age with a histological confirmed leiomyosarcoma or liposarcoma were eligible for enrollment. Any prior treatment was possible except adjuvant chemotherapy. Patients with evidence of newly diagnosed metastatic disease or disease progression during prior chemotherapy within the last six months in computed tomography or magnetic resonance imaging could be included. Patients were required to have a World Health Organisation (WHO) performance status of 0 or 1, an adequate haematological function (haemoglobin ≥9 g/dL, absolute neutrophil count (ANC) ≥ 1.5 × 10^3^/mm^3^, platelets ≥100.000/mm^3^), an adequate hepatic function (serum total bilirubin ≤·upper limit of normal (ULN), serum total ALP ≤ 2.5 × ULN, serum AST and ALT ≤ 2.5 × ULN), and an adequate renal function (glomerular filtration rate (calculated by Cockroft-Gault) ≥ 60 mL/min). The trial was conducted between January 2012 and August 2014.

### 2.2. Study Design and Treatment

The phase I, open-label, dose-escalating combination study was conducted at two GISG centers, the Sarcoma Unit at the Interdisciplinary Tumor Center Mannheim of the Mannheim University Medical Center, University of Heidelberg and the Sarcoma Center Berlin-Brandenburg at HELIOS Klinikum Bad Saarow, Germany. The clinical protocol was approved by the local ethics committees, and all patients provided written informed consent prior to study inclusion. Trabectedin and gemcitabine were administered by intravenous (IV) infusion in a 21-day cycle. Patients were premedicated with IV dexamethason 20 mg prior to trabectedin administration. Gemcitabine was given as a 30-min. peripheral or central infusion on days 1 and 8, followed by a 3-h central infusion of trabectedin on day 1. Planned dose levels were trabectedin 0.7, 0.9, 1.1, 1.3, and 1.5 mg/m^2^ and gemcitabine 700 and 900 mg/m^2^.

### 2.3. Safety

Adverse events (AEs) were graded using the National Cancer Institute Common Toxicity Criteria (NCI-CTC), version 4.0. DLT was defined as any of the following: grade 4 neutropenia (ANC < 0.5 × 10^9^/L) over 5 days, febrile neutropenia (ANC < 1.0 × 10^9^/L over 5 days or with fever [body temperature ≥ 38.5 °C]), platelets < 25 × 10^9^/L, grade ≥ 3 thrombocytopenia accompanied by bleeding, diarrhea ≥ grade 3 despite optimal loperamide use, rash ≥ grade 3, grade 3 transaminases (ALT/AST) increase longer than 7 days duration or grade 4 of any duration and other effects ≥ grade 3 thought to be treatment related (except nausea/vomiting, alopecia grade 2, fatigue lasting less than 48 h, and non-clinically relevant biochemical abnormalities). AEs and toxicity were assessed weekly.

### 2.4. Response Assessments and Statistical Analysis

Treatment responses were determined radiologically every three months based on the Response Evaluation Criteria in Solid Tumors (RECIST version 1.1). All patients who received ≥1 dose of each study drug were included in the toxicity and efficacy analyses. A standard phase I “3 + 3” dose escalation design was used.

## 3. Results

### 3.1. Patients

Five patients received ≥1 dose of study medication and were included in the study analysis population. Relevant patients’ characteristics are summarized in Table 1. Four female and one male patient had a median age at study inclusion of 66 years (range: 50–76). There were four patients with a leiomyosarcoma, three of them of uterine origin, one in the gluteal region; and one patient with a retroperitoneal liposarcoma. All five patients were treated with prior surgery for the primary tumor; two of them received additional adjuvant radiotherapy. Four patients were treated with prior systemic therapy: three of them received first line therapy for metastatic disease consisting of single-agent doxorubicin; one patient was treated with letrozole.

**Table 1:** Patients’ characteristics (*n* = 5).

***Gender***	Female	4
	Male	1
***Age***	Median (years)	66 (range: 50–76)
***Histology***	Leiomyosarcoma	4
	Liposarcoma	1
***Tumor site at initial diagnosis***	Uterus	3
	Abdomen/Retroperitoneum	2
***Metastatic localizations***	Lung	2
	Liver	3
	Abdomen	2
***Previous treatments***	Surgery alone	3
	Surgery plus radiotherapy	2
	Systemic therapy	4

### 3.2. Treatment and Safety

The median duration of combination treatment within the study was 89 days (range: 22–253). The study was stopped due to an unacceptable frequency of DLT in four of five patients. At dose level 1 (trabectedin 0.9 mg/m^2^ plus gemcitabine 700 mg/m^2^), two patients were included and exhibited DLT leading to an amendment of the study protocol enabling the treatment of patients on dose level −1 consisting of trabectedin 0.7 mg/m^2^ and gemcitabine 700 mg/m^2^. An additional two patients experienced a DLT on this dose level. The type of DLT was as follows: two patients demonstrated a platelet count <25 × 10^9^/L on day 15 of the first (patient No. 1) and the fifth treatment cycle (No. 5), respectively; the other two patients (patients No. 2 and 3) stopped treatment due to an increase of transaminases (ALT/AST) grade 3 lasting for more than seven days. An overview of the dose levels, the reason for the end of treatment and the types of DLT is depicted in Table 2. None of these patients had significant clinical problems or symptoms; only one of the patients (No. 1) with thrombocytopenia was hospitalized with fatigue and received a single platelet infusion during hospitalization. This was recorded as one of the three SAEs of the study; the other two SAEs were recorded due to an increased ALT and fever (Table 3). A list of all reported adverse events is depicted in Table 4. No further dose escalation could be performed and, therefore, no maximum tolerated dose could be recommended for phase II, concluding that the combination of gemcitabine and trabectedin is generally not recommended for the treatment of patients with advanced and/or metastatic leiomyosarcoma or liposarcoma.

**Table 2:** Dose levels and Dose-limiting toxicities (DLTs).

Patient No.	Dose Level	Duration of Treatment	Reason for End of Treatment		Type of DLT
			Disease Progression	DLT	
001	1	36 days	no	yes	hematologic
002	1	22 days	yes	yes	non-hematologic
003	−1	30 days	no	yes	non-hematologic
004	−1	253 days	yes	no	-
005	−1	106 days	no	yes	hematologic

**Table 3:** List of all Serious Adverse Events (SAEs).

Patient No.	Dose Level	Type of SAE	DLT	CTCAE-Grade	Related to Study Drug	Action Taken Related to Study Drug	Other Action Taken
1	1	Fatigue	No	2	Possible	Dose not changed	None
2	1	ALT increase	Yes	3	Definite	Dose reduced	Drug treatment
5	−1	Fever	No	1	Unrelated	Not applicable	Drug treatment

**Table 4:** List of all Adverse Events (AEs) *.

	Dose Level 1 (*n* = 2)		Dose Level −1 (*n* = 3)	
Adverse Events	All Grades	Grade 3/4	All Grades	Grade 3/4
WBC decreased	8	5	0	0
Neutrophil count decreased	6	4	0	0
Anaemia	6	0	0	0
Platelet count decreased	6	2	1	1
ALT increased	3	2	8	2
AST increased	2	0	6	1
Alkaline phosphatase increased	1	0	0	0
GGT increased	1	1	0	0
Fever	0	0	13	0
Urinary tract infection	0	0	1	0
Bladder infection	0	0	1	0
Infection ankle bone	0	0	1	0
Skin infection leg	0	0	1	0
Refluxesophagitis	0	0	1	0
Common cold	1	0	0	0
Fatigue	2	0	2	0
Nausea	1	0	1	0
Oedema	0	0	6	0
Circulatory disorder	0	0	1	0
Constipation	0	0	1	0
Pain sternum	0	0	2	0
Troponin T increased	0	0	1	0

Abbreviations: WBC = white blood cells/leucocytes count; ALT = alanin-aminotransferase; AST = aspartat-aminotransferase; GGT = gamma-glutamyltransferase.; * More than one AE may be observed per patient; percentages are not reported due to the low number of patients per dose level.

### 3.3. Efficacy

There were no complete or partial responses in our patient cohort. However, four patients maintained stable disease; so the rate of patients with stable disease is estimated to be 80% at three months and 50% at six months, respectively. Two patients demonstrated prolonged disease stabilization for nine months. One patient (No. 4) with a leiomyosarcoma benefited the longest from the combination treatment, showed no DLT and had to stop study medication due to disease progression nine months after study inclusion.

## 4. Discussion

Standard systemic chemotherapy such as doxorubicin and/or ifosfamide still forms the backbone of palliative treatment in advanced STS. More recently, newer compounds have been added to the treatment armamentarium of STS such as gemcitabine (plus or minus docetaxel), trabectedin and pazopanib. Strategies to improve patients’ outcome are urgently needed; one of these strategies is to combine “targeted” agents with the established chemotherapeutic compounds. Trabectedin gained approval in Europe in 2007 for the treatment of advanced and/or metastatic STS. Administered as single-agent therapy, it has a favorable toxicity profile. The most common side effects such as fatigue and myelosuppression are usually mild. It lacks cumulative and organ-specific toxicity and does not cause many of the problems that have an adverse effect on the quality of life of patients such as severe acute nausea, mucositis, alopecia or skin toxicity. Specific adverse events like transaminitis and rhabdomyolysis have no clinical implications in the majority of patients and are easily manageable [12].

The present GISG phase I trial formally evaluated the combination of trabectedin and gemcitabine in patients with L-sarcomas. Unexpectedly, dose-limiting toxicity occurred on two dose levels so that no further dose escalation could be performed and no recommended dose for phase II testing could be defined. Hence, the results of our trial suggest that the combination of gemcitabine and trabectedin is not recommended for the treatment of patients with advanced and/or metastatic leiomyosarcoma or liposarcoma.

In a phase I trial, the combination of trabectedin and cisplatin given every three weeks in patients with advanced solid tumors has been evaluated [13]. The regimen consisted of cisplatin at a fixed dose of 75 mg/m^2^ 1-h IV infusion followed by escalating doses of trabectedin 3-h IV infusion, both administered on day 1 every 3 weeks. Two DLTs, grade 4 neutropenia longer than 7 days’ duration and grade 3 vomiting despite standard antiemetic therapy, occurred at the starting dose of trabectedin (0.75 mg/m^2^). The immediate lower dose (trabectedin 0.60 mg/m^2^) was evaluated in a total of eight patients; no DLTs occurred and this was declared the recommended dose. The safety profile at this dose and schedule was consistent with the known side effects of each agent alone: nausea, fatigue, transient transaminase elevations and neutropenia; no new or unexpected adverse reactions were observed. The authors concluded that, although the trabectedin dose achieved with this combination was rather low (40% of single-agent when given every three weeks), this combination regimen of trabectedin plus cisplatin was feasible and showed a tolerable safety profile.

To determine the dose of trabectedin plus doxorubicin a phase I combination study was performed in patients with STS treated with 0–1 prior chemotherapy regimen excluding doxorubicin [14]. The regimen consisted of a 10–15-min. IV infusion of doxorubicin 60 mg/m^2^ followed by a 3-h IV infusion of trabectedin 0.9–1.3 mg/m^2^ on day 1 of a 3 week cycle. Because four of the first six patients experienced DLT-defining neutropenia during cycle 1, all subsequent patients received primary prophylactic granulocyte colony-stimulating factor. No DLTs were observed at dose level 1 (trabectedin 0.9 mg/m^2^) and dose level 2 (trabectedin 1.1 mg/m^2^). At dose level 3 (trabectedin 1.3 mg/m^2^) two patients experienced DLTs which were grade 4 neutropenia and grade 4 thrombocytopenia making this dose level unacceptable. The maximum tolerated dose was trabectedin 1.1 mg/m^2^ and doxorubicin 60 mg/m^2^. Common grade 3/4 adverse events were neutropenia (71%), ALT increase (46%), and thrombocytopenia (37%). The authors concluded that the combination of doxorubicin 60 mg/m² followed by trabectedin 1.1 mg/m² every 21 days is safe and active in patients with STS. Promising phase II data in 61 leiomyosarcoma patients demonstrated one complete response, 13 partial responses and 20 stable diseases accounting for a disease control rate of 94%. Common grade 3/4 toxicities were neutropenia (41%), febrile neutropenia (4%), thrombocytopenia (20%), anemia (9%), fatigue (6%), vomiting (4%), and transaminases elevation (9%) [15]. Another phase II study from the Spanish Group for Sarcoma Research (GEIS) evaluating the combination of trabectedin and doxorubicin did not add any further information regarding toxicities [16].

Discussing the phase I/II combination studies, hematologic toxicity (neutropenia and thrombocytopenia) seems to be the major problem when combining trabectedin with cisplatin, doxorubicin or gemcitabine. In combination with cisplatin this DLT led to a rather low dose of trabectedin (only 40% of the approved single-agent treatment dose) questioning the efficacy of this combination. In the combination studies with doxorubicin, hematologic toxicity could be overcome through the use of granulocyte colony-stimulating factor. In our combination trial with gemcitabine, thrombocytopenia was the major limiting factor regarding hematologic toxicity. Notably, both patients with thrombocytopenia were treated previously with adjuvant radiation in the pelvic region which could have had an implication on the occurrence of that type of DLT. Hepatic toxicity was observed quite frequently; however, in the combination with cisplatin and doxorubicin it did not classify for a DLT (grade 3/4 transaminitis lasting ≥7 days). Therefore, prolongation of the elevation of transaminases as observed in our trial seems to be particularly related to the addition of gemcitabine. As described in the literature, gemcitabine may lead to common transient rises in transaminases. However, these laboratory abnormalities are rarely clinically significant [17]. Gemcitabine is also being evaluated in the combination with the anti-angiogenetic agent pazopanib in several phase I and II trials (NCT01593748, NCT01532687, and NCT01442662); however, no data is available yet. Nevertheless, a fatal hepatic serious adverse event occurred in one US study investigating the combination of gemcitabine and pazopanib for the treatment of advanced STS with the consequence to temporarily hold enrolment in all studies of gemcitabine in combination with pazopanib. The fatal hepatic event occurred in a patient with metastatic leiomyosarcoma with multiple liver metastases, with marked elevation of liver enzymes after one dose of gemcitabine and two doses of pazopanib leading to a hepatorenal syndrome and death. Fatal hepatotoxicity is a recognized adverse event associated with both pazopanib and gemcitabine. However, the rapidity of onset and the degree of enzyme elevation in this case is not consistent with the known safety profile of either drug. It seems that gemcitabine prolongs or increases the elevation of liver enzymes significantly.

Taken together, although clinical benefit in the form of disease stabilization was quite promising in our small patient cohort, due to dose-limiting toxicity, the combination of gemcitabine plus trabectedin could generally not be recommended for the treatment of patients with advanced and/or metastatic leiomyosarcoma or liposarcoma. Drug combinations still seem promising, but require careful evaluation in terms of safety.

## 5. Conclusions

The present study of the German Interdisciplinary Sarcoma Group (GISG-02) represents the formal evaluation of the safety and efficacy of trabectedin combined with gemcitabine in so-called L-sarcomas. The study was stopped due to four dose-limiting toxicities in five treated patients. Therefore, the results of our trial suggest that the combination of gemcitabine and trabectedin is not recommended for patients with L-sarcomas. This phase I study strongly supports the necessity for careful evaluation of combination therapies.
